# SAD-B modulates epileptic seizure by regulating AMPA receptors in patients with temporal lobe epilepsy and in the PTZ-induced epileptic model

**DOI:** 10.1590/1414-431X20199175

**Published:** 2020-04-06

**Authors:** Rong Li, Miaoqing He, Bing Wu, Peng Zhang, Qinbin Zhang, Yangmei Chen

**Affiliations:** 1 Department of Neurology, Second Affiliated Hospital of Chongqing Medical University, Chongqing, China; 2 Center for Brain Disorders Research, Capital Medical University, Feng Tai District, Beijing, China; 3 Beijing Institute for Brain Disorders, Feng Tai District, Beijing, China; 4 Department of Neurology, First Affiliated Hospital of Chongqing Medical University, Chongqing Key Laboratory of Neurology, Chongqing, China

**Keywords:** SAD-B, AMPA receptor, Temporal lobe epilepsy, PTZ

## Abstract

α-Amino-3-hydroxy-5-methyl-4-isoxazolepropionic acid (AMPA) receptors are the predominant mediators of glutamate-induced excitatory neurotransmission. It is widely accepted that AMPA receptors are critical for the generation and spread of epileptic seizure activity. Dysfunction of AMPA receptors as a causal factor in patients with intractable epilepsy results in neurotransmission failure. Brain-specific serine/threonine-protein kinase 1 (SAD-B), a serine-threonine kinase specifically expressed in the brain, has been shown to regulate AMPA receptor-mediated neurotransmission through a presynaptic mechanism. In cultured rat hippocampal neurons, the overexpression of SAD-B significantly increases the frequency of miniature excitatory postsynaptic currents (mEPSCs). Here, we showed that SAD-B downregulation exerted antiepileptic activity by regulating AMPA receptors in patients with temporal lobe epilepsy (TLE) and in the pentylenetetrazol (PTZ)-induced epileptic model. We first used immunoblotting and immunohistochemistry analysis to demonstrate that SAD-B expression was increased in the epileptic rat brain. Subsequently, to explore the function of SAD-B in epilepsy, we used siRNA to knock down SAD-B protein and observed behavior after PTZ-induced seizures. We found that SAD-B downregulation attenuated seizure severity and susceptibility in the PTZ-induced epileptic model. Furthermore, we showed that the antiepileptic effect of SAD-B downregulation on PTZ-induced seizure was abolished by CNQX (an AMPA receptor inhibitor), suggesting that SAD-B modulated epileptic seizure by regulating AMPA receptors in the brain. Taken together, these findings suggest that SAD-B may be a potential and novel therapeutic target to limit epileptic seizures.

## Introduction

Epilepsy is a common chronic neurological disease characterized by recurrent spontaneous seizures. It is refractory to current therapies in approximately 30% of patients and is associated with pathologic cortical excitability (1). Temporal lobe epilepsy (TLE), the most prevalent form of refractory epilepsy, representing more than 40% of cases, is intractable (2) and results in serious cognitive impairment, sudden unexpected death in epilepsy (SUDEP), vascular disease, pneumonia, etc. (3). In terms of the pathogenesis of epilepsy, it is widely accepted that an imbalance in neuronal excitation and inhibition results in neurotransmission failure as a causal factor in patients with TLE. α-Amino-3-hydroxy-5-methyl-4-isoxazolepropionic acid (AMPA) receptors, located at the postsynaptic membrane of excitatory synapses, are the predominant mediators of glutamate-induced excitatory neurotransmission in the neural system (4). Dysfunction of AMPA receptors as one of the most important factors in patients with epilepsy has been extensively reviewed (5). Thus, there has been considerable interest in developing therapeutic strategies aimed at AMPA receptors.

Brain-specific serine/threonine-protein kinase 1 (BRSK1, also referred to as SAD-B), a mammalian serine/threonine kinase, is specifically expressed in the brain (6). SAD-B is involved in promoting the maturation of nerve terminals and modulating neurotransmitter release and regulates correct synaptic development and presynaptic vesicle clustering in both sensory and motor neurons (7). Notably, SAD-B associates with synaptic vesicles at the active zone cytomatrix of presynaptic terminals, and the overexpression of SAD-B significantly increases the frequency of miniature excitatory postsynaptic currents (mEPSCs) (8). Therefore, we hypothesized that SAD-B may modulate epileptic seizures by regulating AMPA receptor function in patients with TLE and in the PTZ-induced epileptic model.

In this study, we first observed that SAD-B expression was increased in patients with TLE and in the PTZ-induced epileptic model. Subsequently, we found SAD-B downregulation attenuated seizure severity and susceptibility in the PTZ-induced epileptic model. Furthermore, we found that the effect of SAD-B downregulation on PTZ-induced seizure was abolished by CNQX (an AMPA receptor inhibitor) (9), suggesting that the antiepileptic effect of SAD-B downregulation on PTZ-induced seizure was mediated by AMPA receptors. These results indicated that SAD-B may represent a potential therapeutic target for epilepsy.

## Material and Methods

### Human samples

Samples from twenty patients with TLE (12 males and 8 females; age: 23.35±9.05 years; epilepsy course: 8.35±4.68 years) were obtained from the brain tissue bank of First Affiliated Hospital of Chongqing Medical University. Pre-surgical assessments were thoroughly evaluated to ensure that the patients with epilepsy were suitable for surgery. Twenty histologically normal samples were obtained from patients who experienced head trauma (11 males and 9 females; age: 23.95±8.45 years), did not present with signs of apparent nervous system disease, and were not exposed to antiepileptic drugs (AEDs). The clinical features of the patients included in this study are reported in Tables 1 and 2. No significant difference in sex or age was observed between the two groups (P>0.05). The study protocols were performed according to the guidelines for the implementation of research on humans as established by the National Institutes of Health of China and the Committee on Human Research at Chongqing Medical University. Written informed consent for the use of human brain tissues was collected as described in our previous publication (10).

**Table 1:** Clinical characteristics of patients with temporal lobe epilepsy (TLE).

No.	Gender	Age (years)	Duration (years)	Anti-epileptic drugs	Resected tissue	Pathology result
1	M	7	3	OXC, CLB, VPA, TPM	LTN	G
2	F	12	5	OXC, VPA, GBP	LTN	G, NL
3	F	23	8	OXC, VPA, TPM	RTN	G, NL
4	F	17	5	VPA, CBZ, TPM	RTN	G, NL, ND
5	M	27	5	CBZ, VPA, CLB	LTN	G, NL, ND
6	M	13	7	LTG, TPM, CBZ	RTN	G
7	F	21	4	VPA, PB, CBZ, LEV	LTN	G, NL, ND
8	M	36	18	CBZ, VPA, CLB, TPM	LTN	G, NL, ND
9	M	16	7	OXC, VPA, PHT	LTN	G, NL
10	F	28	11	VPA, CBZ, PHT	RTN	G, NL
11	M	31	15	VPA, CBZ, PHT	RTN	G, NL, ND
12	F	45	20	CBZ, PHT, VPA, PB	LTN	G, NL, ND
13	M	30	7	VPA, PB, CBZ	RTN	G, NL, ND
14	M	25	6	PHT, VPA, PB, TPM	LTN	G, NL, ND
15	M	33	12	CBZ, PHT, LTG	RTN	G, NL, ND
16	M	20	8	CBZ, PB, LTG, LEV	RTN	G, NL
17	M	15	9	LTG, TPM, CBZ	RTN	G, NL
18	F	21	4	VPA, CBZ, PB	LTN	G, NL
19	M	24	5	CBZ, PHT, PB, LTG	LTN	G, NL, ND
20	F	25	8	CBZ, PB, LTG, LEV	RTN	G, NL

F: female; M: male; OXC: oxcarbazepine; CLB: clonazepam; VPA: valproic acid; TPM: topiramate; GBP: gabapentin; CBZ: carbamazepine; LTG: lamotrigine; PB: phenobarbital; LEV: levetiracetam; PHT: phenytoin; LTN: left temporal neocortex; RTN: right temporal neocortex; G: gliosis; NL: neuronal loss; ND: neuronal degeneration.

**Table 2:** Clinical characteristics of the control group.

No.	Gender	Age (years)	Resected tissue
1	F	13	LTN
2	M	24	LTN
3	M	20	RTN
4	M	25	LTN
5	F	25	RTN
6	M	31	LTN
7	M	18	LTN
8	M	44	RTN
9	F	36	LTN
10	F	33	RTN
11	F	22	RTN
12	M	16	RTN
13	M	19	RTN
14	F	27	LTN
15	M	20	LTN
16	F	15	LTN
17	F	9	RTN
18	M	32	RTN
19	F	28	LTN
20	M	22	RTN

All participants had brain trauma, none presented seizure, and pathological results were normal. F: female; M: male; LTN: left temporal neocortex; RTN: right temporal neocortex.

### Experimental animals

Sprague-Dawley rats were obtained from the Laboratory Animal Center of Chongqing Medical University (China). In the chronic model, we administered a sub-convulsive dose of PTZ (35 mg/kg, *ip*, Sigma, USA) daily. The animals were considered to be kindled after exhibiting at least three consecutive stage 4 or 5 seizures (Racine's scale evaluation). In the acute PTZ-induced seizure model, we injected rats with PTZ (70 mg/kg, *ip*) to induce seizures. Diazepam (10 mg/kg) was administered by *ip* injection 1 h after the initial onset of seizures to stop the continuous seizures. All protocols for the handling and treatment of animals were approved by the Commission of Chongqing Medical University for Ethics in Animal Experiments (approval no. 0002648). All experiments were conducted according to the principles of Animal Research: Reporting *in vivo* Experiments (ARRIVE) guidelines (https://www.nc3rs.org.uk/arrive-guidelines). All efforts were made to decrease the number of animals and alleviate their suffering.

### Tissue preparation

All human brain tissues were collected in the operating room as described in our previous publication (10). Minor modifications included the sectioning of the tissue into 30-µm thick sections for double immunofluorescence labelling.

### Immunohistochemistry

Avidin-biotin-peroxidase complex (Vectastain Elite ABC; Vector Laboratories International, USA) was used for immunohistochemistry as described in our previous study (10). Minor modifications included the use of a polyclonal mouse SAD-B antibody (diluted 1:100, Cat. No. ab206298; Abcam, UK) as the primary antibody.

### Double immunofluorescence labelling

Double immunofluorescence staining was conducted on five randomly selected sections as described previously (10). The primary antibodies included mouse anti-SAD-B (diluted 1:100, Cat. No. ab206298; Abcam, UK), chicken anti-microtubule-associated protein 2 (MAP2) (1:200, Cat. No. ab5392; Abcam), and rabbit anti-glial fibrillary acidic protein (GFAP) (1:50, Cat. No. BM0055; Boster, China). The secondary antibodies included Alexa Fluor 488-conjugated goat anti-rabbit IgG (1:100; Proteintech, China), DyLight 405-conjugated goat anti-chicken IgG (1:50, Beyotime Institute of Biotechnology, China), and Alexa Fluor 549-conjugated goat anti-mouse IgG (1:100; Proteintech, China).

### Immunoblotting

Human brain tissues from patients with TLE or traumatic brain injury, as well as rat cortices and hippocampi, were homogenized in RIPA lysis buffer (Proteintech, China). The supernatants were removed after centrifugation at 4°C (12,000 *g*, 15 min). Prepared proteins (40 μg per lane) were separated by SDS-polyacrylamide gel electrophoresis (SDS–PAGE; 5% spacer gel, 80 V, 30 min; 10% separating gel, 120 V, 90 min) before being transferred to a polyvinylidene fluoride (PVDF) membrane (220 mA, between 60 and 120 min according to the molecular weight of the protein). Next, the PVDF membrane was incubated at 37°C for 1 h in 10% skim milk in Tween-20 for blocking. The PVDF membrane was then incubated with polyclonal mouse anti-SAD-B-1 (diluted 1:100, Cat. No. ab206298; Abcam) diluted in 10% skim milk diluted in freshly prepared 5% BSA at 4°C overnight. The membrane was washed with Tween-20-Tris-buffered saline (TBS) for 45 min, with the solution changed every 10 min, and then incubated with goat anti-mouse IgG antibody or goat anti-rabbit IgG antibody (1:1000, Proteintech, China) for 1 h at 37°C. A rabbit anti-GAPDH antibody (1:1000, Proteintech) was used as the loading control. Densitometry quantitation was determined using Quantity One 1-D Analysis Software (Bio-Rad Laboratories, USA) as optical density values, and the SAD-B levels were normalized to those of GAPDH.

### Co-immunoprecipitation

Human cortical and rat hippocampal tissues were homogenized and mixed with RIPA lysis buffer (Beyotime, China). Equal amounts of protein were then incubated with antibody (4 μL of polyclonal mouse anti-SAD-B1 (1:50 Cat. No. ab206298; Abcam, UK), 4 μL of mouse anti-GluA1–GluA4, (1:100, Santa Cruz Biotechnology, USA) or 2 μL of rabbit IgG, which served as a control (1:100, Abcam). The supernatant fractions were incubated for 12 h at 4°C and then incubated with 15 μL of protein A/G agarose beads (Beyotime) for 1.5 h at 4°C. The immunoprecipitates were washed twice with lysis buffer, boiled in 1× SDS loading buffer for 10 min and separated on SDS-polyacrylamide gels. Immunoblotting experiments were performed with anti-GluA1-GluA4 and anti-SAD-B antibodies.

### Behavioral tests

Two observers who were blinded to the handling conditions were responsible for recording the general behaviors induced by PTZ. Seizures were induced between 8:00 and 10:00 am to minimize the possible complicating effects of the animals' circadian rhythms. For seizure severity, the behaviors were scored as follows (11): 0) no further seizures; 1) a generalized clonic seizure; 2) a generalized clonic seizure with loss of righting reflex; 3) a generalized clonic seizure with loss of righting reflex plus running and bouncing; and 4) all behaviors associated with a score of 3 plus forelimb tonus. Latency was recorded as the time after PTZ injection to the first seizure onset. The generalized tonic clonic (GTC) value per rat is the number of GTCs per rat within 10 min of PTZ injection.

### Lentiviral vector generation and stereotaxic injection

Lentiviral (LV) vectors carrying siRNA were constructed by a ubiquitin promoter (GeneChem Co., Ltd., China). The control (no target siRNA) was the same lentiviral vector expressing GFP (LV-GFP). We performed microinjections of siRNA or LV-GFP into the bilateral CA1 region of the dorsal hippocampus of each animal in a total volume of 20 μL (10 μL per side) using a glass pipette (0.2 μL/min). Two weeks after the administration of the lentiviral vectors, we induced seizures with PTZ.

### *In vivo* multichannel EEG recording and local field potential (LFP) analysis

After SAD-B-siRNA was injected into the CA1 region (AP –3.6 mm, ML –2.6 mm, and DV –2.8 mm), a microwire array (a 1.5×6 array of platinum-iridium alloy wires, each with a 20-μm diameter) was implanted into the same site in the CA1 region of the hippocampus. PTZ was administered to induce seizures (after two weeks). *In vivo* multichannel EEG recordings were continuously collected for 10 min after the administration of PTZ. According to the PTZ scoring criteria, an electrophysiological seizure was defined as the manifestation of seizure behaviors ranging from stages 1–4 in the rats. The intra-hippocampal LFP of electroencephalography (EEG) recordings showed a high-amplitude discharge (>3-fold higher the baseline) that began in the hippocampus and spread to the cortex with a high frequency (>5 Hz). Latencies and generalized tonic clonic seizures (GTCs) in the observed 10 min period during status epilepticus were recorded for each animal from each group. LFPs were preamplified (1000×), filtered (0.1–1000 Hz), and digitized at 4 kHz using an OmniPlex D Neural Data Acquisition System (Plexon, USA). All animals were euthanized with pentobarbital (100 mg/kg, *ip*, Sigma) after the seizures were recorded and hippocampal tissues were collected for morphological and biochemical studies.

## Results

### SAD-B was localized in the epileptic brain

**Figure 1:**
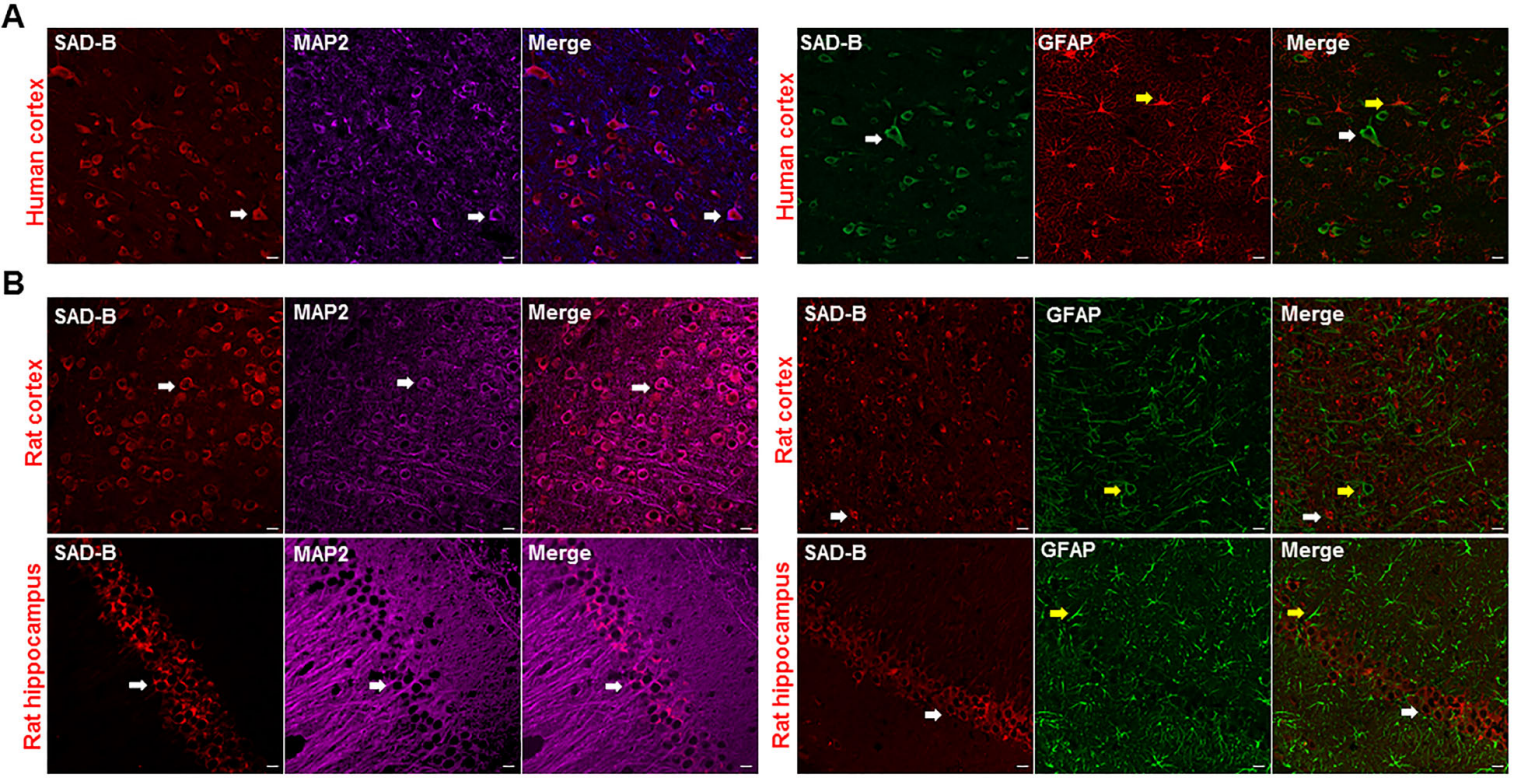
Brain-specific serine/threonine-protein kinase 1 (SAD-B) is localized in the epileptic brain. **A**, Immunofluorescence labelling of SAD-B (red), MAP2 (violet), and GFAP (green) in the cortex of patients with temporal lobe epilepsy (TLE) showing that SAD-B was co-localized with MAP2 but not with GFAP. Scale bar: 50 μm (400×). **B**, Immunofluorescence labelling of SAD-B (red), MAP2 (violet), and GFAP (green) in the CA1 region of the hippocampus or cortex of an epileptic rat showing that SAD-B was co-localized with MAP2, but not with GFAP. Scale bar: 50 μm (400×). White arrows: SAD-B; yellow arrows: GFAP.

Epilepsy is known to be characterized by neuronal hyperexcitability, thus, we first determined whether SAD-B is located in neurons. Immunofluorescence showed that SAD-B was localized in the cortical neurons of the patients with TLE (Figure 1A). In the PTZ-induced epileptic model, SAD-B was localized in the cortex and hippocampus of epileptic rats (Figure 1B). SAD-B was co-expressed with the neuron-specific marker MAP2 in neurons but not with the astrocyte-specific marker GFAP.

### SAD-B expression was increased in the epileptic brain

**Figure 2:**
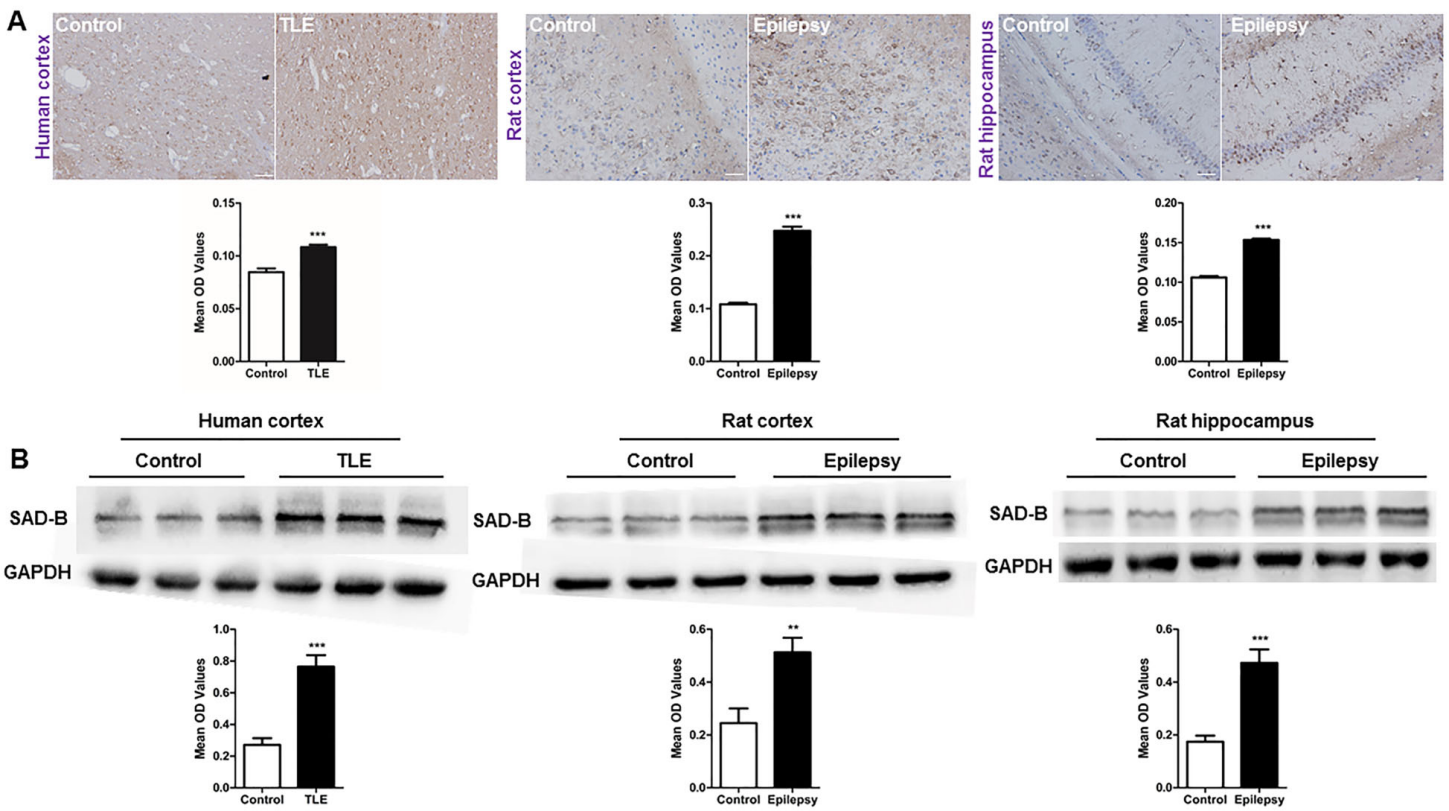
Brain-specific serine/threonine-protein kinase 1 (SAD-B) expression is increased in the epileptic brain. **A**, Immunohistochemistry for SAD-B (left panels) showing that the average optical density (OD) of SAD-B in patients with temporal lobe epilepsy (TLE) was increased compared to that in the controls (n=5 pairs, ***P<0.05, unpaired *t*-test). In the epileptic rats, SAD-B expression was also increased in the cortices and hippocampi of PTZ-induced epileptic rats compared to that in the controls (n=5 pairs, ***P<0.05, unpaired *t*-test). Scale bar: 100 μm (400×). **B**, Immunoblot analysis showing that SAD-B expression was significantly increased in patients with TLE compared to controls (n=12 pairs, ***P<0.001, unpaired *t*-test). In the epileptic rats, SAD-B expression was also significantly increased in the cortices and hippocampi of the PTZ-induced rats compared to controls (n=12 pairs, **P<0.01, ***P<0.001, unpaired *t*-test). Data are reported as mean±SEM.

To examine whether SAD-B is involved in epileptic seizure, we examined SAD-B expression in the patients with TLE. Immunohistochemistry (IHC) showed that SAD-B expression was significantly increased in patients with TLE compared to the controls. Moreover, immunoblotting was performed and the results showed that SAD-B expression was also significantly increased in patients with TLE (Figure 2A). In epileptic rats, SAD-B expression was increased in the cortex and hippocampus compared to the controls (Figure 2B). These findings indicated that SAD-B may be involved in epileptic seizure in the brain.

### SAD-B downregulation reduced seizure severity and susceptibility in the PTZ-induced model

**Figure 3:**
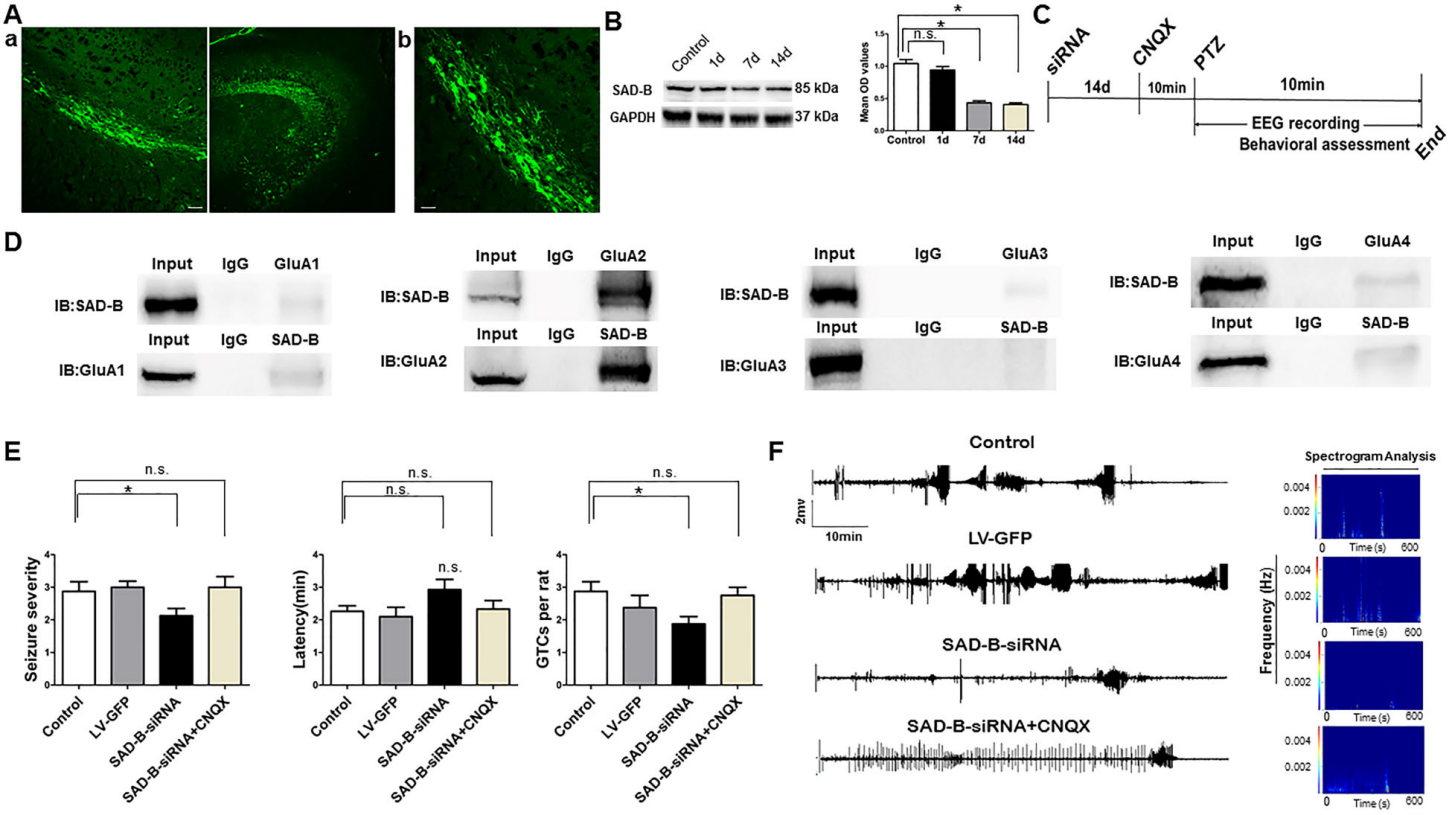
The antiepileptic effect of brain-specific serine/threonine-protein kinase 1 (SAD-B) downregulation is mediated by AMPA receptors. **A**, Fluorescence (FITC)-labelled cells in the hippocampus of the rat brain showed fluorescent neurons that were transfected with exogenous LV-GFP. **a**: Scale bar: 50 µm (200×). **b:** Scale bar: 50 µm (400×). **B**, Immunoblot analysis showing that SAD-B expression was significantly decreased in the hippocampus 7 and 14 days after SAD-B-siRNA injection compared to controls (n=4 in each, *P<0.05, one-way ANOVA). **C**, Schematic overview of SAD-B-siRNA+CNQX administration *in vivo*. **D**, Representative western blotting images of co-immunoprecipitation showing a positive interaction between SAD-B and GluA2 in the hippocampi of epileptic rats. **E**, Behavioral evaluations showing that seizure severity and GTCs per rat were reduced in the SAD-B-siRNA group compared with the control group and that the latency was prolonged in the SAD-B-siRNA group compared to the control group (n=8 in each, *P<0.05). However, seizure severity and GTCs per rat were not reduced in the SAD-B-siRNA+CNQX group compared to the control group, and the latency was not prolonged in the SAD-B-siRNA+CNQX group compared to the control group, suggesting that the antiepileptic effect of SAD-B downregulation was abolished by CNQX. Statistical significance was evaluated by one-way ANOVA. If the variance was homogeneous, the LSD method was used to make multiple comparisons of the mean of each group; when the variance was not homogeneous, the Kruskal-Wallis test and Dunnett's T3 method were used. **F**, Hippocampal EEG recordings and spectrogram analysis of the electrographic results showing that seizure severity and susceptibility were less intense in the SAD-B-siRNA group than in the control group; this effect was abolished by CNQX. Data are reported as mean±SE. n.s., not significant.

Subsequently, to explore the function of SAD-B in epileptic seizure, we downregulated SAD-B expression and observed the behavior of the PTZ-induced epileptic animals. We administered SAD-B-siRNA into the hippocampus of the animal models and observed behavior. After the injection of the exogenous recombinant lentivirus, we first detected the effect of siRNA transfection in the hippocampus 14 days later. We visualized GFP-positive cells in the hippocampus 14 days later in control animals as well (Figure 3A). Moreover, immunoblotting showed that SAD-B expression was significantly decreased in the hippocampus 7 days and 14 days after the initial injection (Figure 3B), indicating that the recombinant lentivirus was successfully transfected into the neurons in the hippocampus. Fourteen days after the administration of the lentiviral vector, we evaluated behavior following PTZ-induced seizures. Seizure severity and susceptibility were significantly reduced (lower seizure severity, fewer GTCs per rat) in the SAD-B-siRNA group compared to the control group (Figure 3E). Furthermore, multichannel hippocampal EEG recordings showed that the seizure severity and susceptibility were less intense in the SAD-B-siRNA group than in the control group (Figure 3F). These findings indicated that SAD-B was involved in epileptic seizure and that SAD-B downregulation can attenuate seizure severity and susceptibility in the PTZ-induced epileptic model.

### The antiepileptic effect of SAD-B downregulation was mediated by AMPA receptors

SAD-B regulates AMPA receptor-mediated neurotransmission through a presynaptic mechanism (8). Thus, we explored the possibility that this mechanism is involved in epilepsy. We found that SAD-B interacted with GluA2 (AMPA receptors are comprised of four subunits (GluA1-GluA4) (Figure 3D). Animals were treated with siRNA or vehicle (14 days prior to receiving PTZ), and then with CNQX (0.5 μg/kg, 10 min prior to receiving PTZ). However, when AMPA receptors were blocked by CNQX, SAD-B downregulation no longer attenuated seizure severity and susceptibility in the PTZ-induced model (Figure 3E). Moreover, multichannel hippocampal EEG recordings also showed that the seizure severity and susceptibility were not attenuated in the SAD-B-siRNA+CNQX group compared to the control group (Figure 3F). These findings indicated that the antiepileptic effect of SAD-B downregulation in PTZ-induced epilepsy was likely mediated through AMPA receptors.

## Discussion

Epilepsy is one of the most common neurological disorders in the nervous system and its pathophysiological alterations result in abnormal discharges characterized by the periodic and unpredictable occurrence of seizures. Epilepsy has many causes (e.g., genetic, developmental, and acquired), each of which may lead to abnormal neuronal synchronization. Cascading excitation within networks of synaptically connected excitatory glutamatergic neurons plays a major role in epilepsy (4). AMPA receptors, the predominant mediators of glutamate-induced excitatory neurotransmission, mediate fast neurotransmission at the postsynaptic site. AMPA receptors are critical for the generation and spread of epileptic activity (4), and evidence has illustrated that AMPA receptors show hypersensitivity in human hippocampal and neocortical tissue in the brains of patients with epilepsy (12,13). The upregulation of AMPA receptors is associated with audiogenic seizures and pharmacoresistant focal temporal lobe epilepsy (14,15). In an epileptic animal model, AMPA receptor phosphorylation was shown to be involved in a pilocarpine model of epilepsy, and alterations in AMPA receptor binding and subunit mRNA expression leads to generalized PTZ-induced seizures (16,17). Notably, evidence has shown that AMPA receptor agonists initiate seizures and that AMPA receptor antagonists induce antiepileptic activity (18). Thus, AMPA receptor blockade was applied, and it should have a promising therapeutic anticonvulsant potential.

SAD-B belongs to the AMPK-related subfamily of protein kinases and contains a conserved kinase domain (KD), an adjacent ubiquitin-associated (UBA) domain, and a kinase-associated domain 1 (KA1) (19). In this study, we demonstrated that SAD-B is involved in epileptic seizure by regulating AMPA receptor function in the PTZ-induced epileptic model. We first identified that SAD-B is located in the cortex and hippocampus of epileptic rats, in which hippocampal sclerosis is the most important pathological characteristic of epilepsy (20,21). This result was consistent with a previous study that indicated that SAD-B is localized in cultured rat primary hippocampal neurons (22). Subsequently, we showed that SAD-B expression was increased in patients with TLE, which indicated that SAD-B may be involved in epilepsy. To further explore the mechanism underlying this alteration and the role of SAD-B in epilepsy, we established the PTZ-induced model, which is the most widely accepted animal model for studying the effects of novel antiepileptic molecules (11). We found that SAD-B expression was also increased in the PTZ-induced model, which is consistent with the results in patients with TLE. These findings suggested that SAD-B may exhibit correlations with epileptic seizure. In nervous system diseases, there is evidence that SAD-B plays multiple sequential roles in neurons, including neuronal polarization, the control of axonal arborization, differentiation, specification, and the promotion of the maturation of nerve terminals (6,8,22–26). SAD-B mutants limit the localization of synaptic vesicle proteins to axons, organize them at synapses, and regulate synaptic vesicle distribution and the development of normal synapses in *C. elegans* (7,27). Tsc1/Tsc2 promotes axonal growth via the upregulation of SAD kinase in tuberous sclerosis complex, which is characterized by tumor predisposition and neurological abnormalities, including epilepsy, mental retardation, and autism (28). SAD-B knock-out mice exhibit defects in neuronal polarity and die 2 h after birth; the embryos show visibly thinner cortices and their neurons lack distinct axonal and dendritic processes (23 [Bibr B24] [Bibr B25]). Most importantly, SAD-B has been characterized as a presynaptic kinase that associates with synaptic vesicles at the active zone cytomatrix of presynaptic terminals and regulates presynaptic AMPA release (8). In this study, we showed that SAD-B blockade attenuated PTZ-induced epileptiform seizures and that the anticonvulsant effect of SAD-B blockade was mediated by AMPA receptors.

Using co-immunoprecipitation, we found that SAD-B interacted with GluA2, which is one of the subunits of AMPA receptors in the brain. Its interaction with GluA2 suggests that it may cause epilepsy. In the nervous system, 90–95% of AMPA receptors are GluA1/GluA2 heteromers (29,30). Moreover, approximately 80% of synaptic AMPA receptors in the CA1 region of the hippocampus are GluA1/GluA2 heteromers ([Bibr B29] [Bibr B30] 31). Increasing evidence has indicated that GluA2 expression is reduced in a model of status epilepticus and acquired epilepsy (32). Furthermore, in pilocarpine-induced SE, the GluA2 reduction may last for several weeks (33,34). GluA2-lacking AMPARs play an important role in the enhancement of responses to Glu in epileptic patients (35). Therefore, the GluA2 heteromers constitute the most prominent AMPA receptors in pyramidal synapses (36). They mediate fast excitatory synaptic neurotransmission in the nervous system and play an essential role in synaptic plasticity (37).

Drug treatment is ineffective for many patients and surgical treatment is only performed if the benefits outweigh the risks (such as hippocampal sclerosis). For these reasons, new treatments complementary to pharmacological therapy need to be developed. AMPA receptor blockade should have a promising therapeutic anticonvulsant potential (4,38). In this study, we showed that SAD-B downregulation attenuated seizure severity and susceptibility by regulating AMPA receptors in patients with TLE and in the PTZ-induced model. The role of SAD-B in the regulation of AMPA receptor function indicated that SAD-B may be a desirable target for developing therapeutic strategies aimed at epilepsy. However, the exact molecular mechanism of the SAD-B signaling pathway in seizure progression needs to be further elucidated. Whether SAD-B is involved in seizure progression through other processes, such as controlling axonal arborization and promoting the maturation of nerve terminals, needs to be further explored.
