# Erratum notice for: “SAD-B modulates epileptic seizure by regulating AMPA receptors in patients with temporal lobe epilepsy and in the PTZ-induced epileptic model” [Braz J Med Biol Res (2020) 53(4): e9175]

**DOI:** 10.1590/1414-431X20209175erratum

**Published:** 2020-09-07

Rong Lihttps://orcid.org/0000-0002-4465-3324 ^1^, Miaoqing Hehttps://orcid.org/0000-0001-7116-0982 ^2,3^, Bing Wuhttps://orcid.org/0000-0002-7153-4136 ^4^, Peng Zhanghttps://orcid.org/0000-0001-8071-3729 ^1^, Qinbin Zhanghttps://orcid.org/0000-0002-9772-1511 ^1^, and Yangmei Chenhttps://orcid.org/0000-0002-0560-8299 ^1^

^1^Department of Neurology, Second Affiliated Hospital of Chongqing Medical University, Chongqing, China

^2^Center for Brain Disorders Research, Capital Medical University, Feng Tai District, Beijing, China

^3^Beijing Institute for Brain Disorders, Feng Tai District, Beijing, China

^4^Department of Neurology, First Affiliated Hospital of Chongqing Medical University, Chongqing Key Laboratory of Neurology, Chongqing, China

Correspondence: Yangmei Chen: <chenym1997@cqmu.edu.cn>

**Erratum for:** Braz J Med Biol Res | doi: 10.1590/1414-431X20199175 | PMID: 32267308 | PMCID: PMC7162585

On July 7, 2020, the Brazilian Journal of Medical and Biological Research received a request from the first author Rong Li requesting the substitution of Figure 1B, Panels: Rat Hippocampus SAD-B, MAP2, and MERGE because these 3 images had been submitted incorrectly. This modification of Figure 1 does not change the findings of this research. After careful evaluation by the Editors, this erratum is being published.

**Figure 1:**
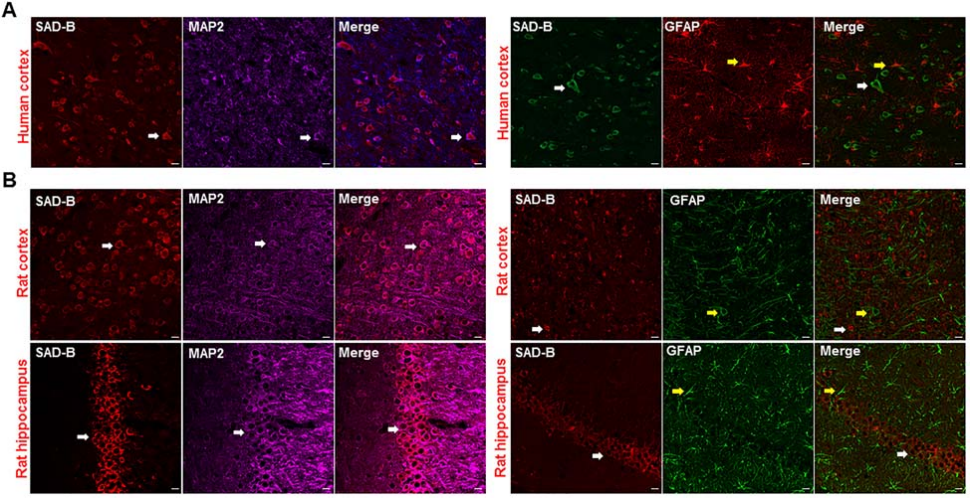
Brain-specific serine/threonine-protein kinase 1 (SAD-B) is localized in the epileptic brain. **A**, Immunofluorescence labelling of SAD-B (red), MAP2 (violet), and GFAP (green) in the cortex of patients with temporal lobe epilepsy (TLE) showing that SAD-B was co-localized with MAP2 but not with GFAP. Scale bar: 50 μm (400×). **B**, Immunofluorescence labelling of SAD-B (red), MAP2 (violet), and GFAP (green) in the CA1 region of the hippocampus or cortex of an epileptic rat showing that SAD-B was co-localized with MAP2, but not with GFAP. Scale bar: 50 μm (400×). White arrows: SAD-B; yellow arrows: GFAP.

